# Effects of early marriage among women married before reaching 18 years old (qualitative study approach)

**DOI:** 10.3389/fsoc.2024.1412133

**Published:** 2024-11-15

**Authors:** Shiferaw Gelchu Adola, Dessalegn Wirtu

**Affiliations:** ^1^Department of Nursing, Institute of Health, Bule Hora University, Bule Hora, Ethiopia; ^2^Department of Public Health, College of Health and Medical Science, Wollega University, Nekemte, Ethiopia

**Keywords:** effects of early marriage, consequences, women, Ethiopia, qualitative study

## Abstract

**Background:**

The consequences of early marriage have become a global concern for young women. The detrimental effects of early marriage disproportionately affect girls. Regarding its effects in poor nations like Ethiopia, there is a dearth of data. As a result, this study offers baseline, first-hand, and updated information for both the nation and the study area.

**Objective:**

The purpose of this study was to investigate the effects of early marriage on Guji women who were married before the age of 18.

**Methods:**

This qualitative study used a phenomenological approach and was carried out from January to April 2024. An in-depth interview technique was employed to collect the data. The interviews were conducted in the Oromo language. A purposive sampling technique was applied to choose the study participants. A total of 25 women who met the eligibility criteria were selected. A thematic data analysis approach was employed to analyze the data in this study.

**Results:**

Three themes, 10 subthemes, and more than hundreds of codes were extracted after the interview data were analyzed. The main themes and subthemes of this study were as follows: 1-Precipitators of early marriage (abduction and arranged marriage); 2-Consequences of early marriage (emotional damage, moral damage, early marriage is a risk factor for maternal and child health, early marriage is a major cause for girls to drop out of school, poverty, gender-based violence and divorce, lack of essential life skills); and 3-Attitudes toward early marriage (early marriage is a harmful cultural practice; girls should be protected).

**Conclusion:**

The study’s findings illustrated the multidimensional effect of early marriage, necessitating the involvement of numerous sectors and stakeholders. The community, religious leaders, the health, education, and legal sectors, as well as the parents of young girls, should all take an active role in delaying early marriage. This indicates the development of policies that permit cooperation amongst all relevant parties. Creating awareness among girls, families, and the community through community-based education is crucial. Early married women should participate in intervention programs to provide their experience to young girls. Women who are victims of early marriage must receive full support to mitigate its negative consequences.

## Introduction

Marriage is a ritual or process that permits a couple’s legal partnership as husband and wife (Haarr and Duncan, 2023).It is the foundation of a family, which is a social institution that grants children legitimacy, gives partners rights and responsibilities, and creates a framework for procreation (Leach, 1955). In other words, early marriage occurs when one or both of the partners are under the age of 18 (Haarr and Duncan, 2023). Early marriage has numerous effects (consequences) on women’s lives across various aspects. These include poor maternal and child health outcomes, lower level of education, gender-based violence, and poverty (Haarr and Duncan, 2023; OHCHR, 2019).

Prior studies revealed an increase in the risk of sexual transmitted diseases (STDs), obstructed labor, obstetric fistulas, premature birth, maternal and newborn mortality, and other pregnancy-related problems, risk of recurrent physical ailments, depression, psychological anguish, pregnancy termination, and a brief period between births (Seta, 2023; Nour, 2006; Yoosefi Lebni et al., 2023; Elnakib et al., 2022).

Furthermore, early marriage interferes with the utilization of health care services. Research indicated that women who have married under the age of 18 had a lower likelihood of giving birth in health care facilities, and less utilizing antenatal and postnatal care (Elnakib et al., 2022; Li et al., 2021; Sekine and Carter, 2019).

Similarly, early marriage is a barrier to education and job opportunities. School dropout, lower level of education, inability to finish primary school, failure to develop intellectual capacity, lack of career opportunity, economic dependence, and poverty were related to underage marriage (Seta, 2023; Yoosefi Lebni et al., 2023; Bengesai et al., 2021; Mim, 2017; Dahl, 2010). Likewise, women married before the age of 18 were more likely to experience intimate partner violence, disputes with their spouses, and marital breakups (Abera et al., 2020; Kidman, 2017).

Underage marriage violates girls’ fundamental human rights by forcing them to leave their families, marry against their will, suffer both physical and sexual abuse from assault, and have children when they are still in adolescence (Vogelstein, 2013). According to research, girls who married at young age face several adverse consequences, including a loss of autonomy, harsh control, a high degree of responsibility, and social isolation (Yoosefi Lebni et al., 2023; Baysak et al., 2021).

Research indicates a robust correlation between fertility rates and early marriage (Yaya et al., 2019).Throughout their reproductive lives, women who marry before the age of 18 have more children than those who marry after the age of 18 (Yaya et al., 2019). Underage marriage and early childbirth have substantial financial impacts on national and family budgets (Wodon et al., 2017). Countries are becoming more difficult to pay for the public spending required to alleviate poverty, hunger, and malnutrition, as well as to guarantee that everyone has access to health care, education, and other necessities as a result of population growth (Wilmoth et al., 2022).

A report by the United Nations Children’s Fund (UNICEF) states that more than 650 million women and girls alive today were married before turning 18 (UNICEF, 2022). A sizeable 54% of women in Sub-Saharan Africa were married at a young age (Yaya et al., 2019). Among them, Eastern and Southern Africa is home to about 32%. With 17.3 million early-married women, Ethiopia is the leading country in the region (UNICEF, 2022).

There are many reasons why girls marry at a young age worldwide. In Turkey, the main causes of early marriage were not having an opportunity for education, the loss of parents, and an unfavorable home environment (Baysak et al., 2021). Early marriage is seen as a cultural tradition in Iraq that has been passed through generations. It is viewed as a tool for regulating girls’ sexual behavior and avoiding extramarital affairs. Additionally, the primary drivers of early marriage were gender inequity and the belief that marriage was a solution to the problem (Hosseini and Asadisarvestani, 2022). Peer pressure plays a significant role in Ethiopia’s tradition of accepting first marriage proposals, which are considered auspicious (Tewahido et al., 2022). Other causes of underage marriage include protecting a girl’s virginity, fear of marital issues if girls grow older, and building closer links between families through girl exchanges (Alemu, 2008).

Early marriage is a worldwide public health concern that is planned to be eradicated by 2030 as one of the sustainable development targets (United Nations, 2015). Globally, the practice of early marriage has decreased over the past two decades. To meet the SDG targets, however, a 20-fold faster rate of reduction is required (UNICEF, 2023).

Examining the impact of early marriage is essential to averting its numerous detrimental effects.

The low-cost solutions for preventing underage marriage include; preventing girls from dropping out of school, giving them better work possibilities, raising awareness of the use of modern contraceptives, strengthening the law’s prohibition of marriages under the age of 18, preventing early pregnancies, and delivering appropriate training and psychological counseling (UNICEF, 2021; Yoosefi Lebni et al., 2023).

The Ethiopian government also pledged to end early marriage by 2025 (UNICEF, 2018). Moreover, marriages under the age of 18 are strictly forbidden under FDRE constitution article 35 (Constitution, 1995). Although the frequency of early marriages has decreased over the past 20 years, many girls across all regions are still at risk of marrying at an early age (Alem et al., 2020; Ethiopia FDRo, 2019).

Moreover, there is a dearth of data regarding the impact of early marriage on women, communities, and the nation as a whole. Therefore, little is known about the effects of underage marriage in the research area or nationwide. This study focused on examining the impact of early marriage to support women who are victims of early marriage and involving them in teaching young females through real-life examples.

## Methods

### Study setting

The study was carried out in the West Guji zone, Southern Oromia, Ethiopia. It is located 470 km away from Addis Ababa, the capital city of Ethiopia. The Gedio zone of Southern Ethiopia and the Sidama regional state border the zone to the north, the Borana zone to the south, the Guji zone to the east, and the South Ethiopia region to the west. According to Ethiopia’s 2007 national census, the zone is home to 1.424,267 million people, of which 1,318,824 live in rural areas and 105,443 in urban areas (Agency, 2007). Guji society is one of the Oromo ethnic groups and speaks the Afan Oromo language. They regularly practice the Gada system, which is a traditional Oromo culture. Guji Oromo and other Oromo ethnic groups, especially Borana, have similar lifestyles (Mulleta and Muda, 2021).

This study was conducted from January to April 2024.

### Study design

This study used a qualitative, phenomenological approach to examine the effects of early marriage on the lives women who marry before turning 18. The phenomenological approach is a study methodology that endeavors to elucidate the fundamental essence of phenomena as perceived by the individuals involved. This method is used to explore encounter from a particular phenomenon or life event (Teherani et al., 2015).

### Study participants and sampling technique

The study participants were women who were married age younger than 18 years and who satisfied the inclusion criteria. The inclusion criteria were women who were married before the age of 18, who were currently under the age of 30, who had resided in the West Guji zone for more than 6 months, who had been married for less than 10 years, and who desired to participate in the study. The study participants were chosen using purposeful sampling techniques. Initially, participants who met the inclusion criteria were identified by health extension workers and the places for interviews were adjusted. The health extension workers identified 30 eligible women in total during the study period, and 25 interviews resulted in data saturation.

### Data collection

The approach used to obtain the data was face-to-face in-depth interviews. The interviews were conducted from January to April 2024. A researcher with experience in gathering qualitative data performed the interviews. Health extension staff worked with us to set up the interview settings. All participants were informed of the study’s purpose before the onset of the interview. Then, written and verbal informed consent was obtained to participate in the study and record their voice, respectively. Interview guide instruments and an audio recorder were utilized. To obtain comprehensive information, this interview used open-ended questions. The primary author transcribed the interview verbatim, which was conducted in Afan Oromo, the official working language of the Oromia region. First, insensitive inquiries concerning sociodemographic characteristics such as present age, marital age, marital status, educational attainment, and number of children were asked. Subsequently, important inquiries to explore effects of early marriages followed. The interview questions covered many issues, including who is engaged in the marriage, their feelings, their attitudes about the marriage, if they faced negative effects from their marriage, and how the effects affected their lives. Probing inquiries were raised whenever needed. Each interview lasted 45–60 min to collect rich, in depth, and comprehensive data. The nonverbal cues during the interviews were also documented, in addition to their voices. As long as new concepts emerged from the interviews, the data collection process continued.

### Data analysis

In this study, thematic data analysis was performed. It is a method for finding, examining, and summarizing themes in data (Braun and Clarke, 2006). The advantage of this method is its flexibility and adaptability for data analysis in many types of qualitative studies (Braun and Clarke, 2006). After hearing the audio several times, the primary author initially typed out a literal transcript of the material in Microsoft Word. The primary author subsequently translated the transcription into English. After qualitative research experts reviewed the translated data, suggestions were taken into consideration. Afterward, a six-phase approach for thematic analysis was implemented;

First, to become acquainted with the data, the transcripts were read repeatedly and audio listened attentively. In this phase, similarities and differences within the dataset were identified. Second, to extract initial codes, the main points were determined once familiarities with the data were confirmed. During this stage, codes were created using Atlas.ti 9 (2021). Third, an analysis was conducted to compare and contrast the codes. Candidate themes and subthemes were identified by grouping similar codes together.

Fourth, to ensure that the themes accurately reflected the dataset, the candidate themes were examined and improved as they captured all codes within their categories. Fifth, themes were established and nominated. To prevent themes from overlapping, the main ideas of every candidate theme and the aspects of the data they captured were determined. Following the definition and nomination of the themes and subthemes, the report was written in accordance with the guidelines for reporting qualitative research (Tong et al., 2007).

### Trustworthiness

To ensure the validity and reliability of the study, the four criteria suggested by Lincoln and Guba, credibility, transferability, dependability, and conformability were applied (Lincoln and Guba, 1985). Extended periods of fieldwork and member checking were conducted to validate the credibility of the research. A thick description was applied to ensure transferability. To ensure external validity and provide a sufficient description of the impact of underage marriage, rich, detailed data covering all experiences of women about early marriages were gathered. Dependability was attained by inviting two researchers who were not involved in the study. Disagreements were resolved when the two researchers independently reviewed all study procedures and outcomes. Furthermore, the processes and study results were accepted by the researchers. Finally, the study’s conformability was preserved by directly quoting participant statements and ensuring that the researcher’s opinions and predetermined judgments were kept out of the study.

### Ethical consideration

Ethical guidelines were followed in this study. First, the Hule Hora University Institutional Review Board Committee approved this study prior to its start (Ref.no.BHU.SGS.213. 4,005). Subsequently, women who fulfilled the research eligibility requirements were identified and invited to participate in the study by health extension workers. Participants’ contact information, the time to participate in the study, and the locations of the interviews were also shared.

Before the interview, each participant received clarification regarding the nature, advantages, and objectives of the study. Next, they signed an informed consent form and gave verbal approval for their voices to be recorded. For privacy, the interviews were held in a separate room. To ensure their anonymity, all research participants were given pseudonyms. A code and password were used to protect the confidentiality of the data that was gathered and kept in electronic files.

No study participants under the age of 18 participated in the present study. All procedures used in this study complied with the ethical standards of the Declaration of Helsinki.

## Results

Twenty-five women who were married before 18 years old participated in this study. The mean age of the participants was 23.64 (±2.39 SD), and the mean age at marriage was 15.84 (±0.85 SD). Twenty-two interviewed women were in a relationship with their husbands, whereas three of them were divorced. Concerning the level of education, the majority of interviewees attended elementary school (grades 1–6). Additional sociodemographic characteristics of the study participants are displayed in Table 1.

**Table 1:** The sociodemographic attributes of the 25 women who were interviewed.

Characteristics	Frequency (*n*)	Percent (%)
Current age of the participants		
≤ 20	4	16
20–25	16	64
>25	5	20
Marriage age of the participants		
14	1	4
15	8	32
16	10	40
17	6	24
Marital status of the participants		
In union with house bands	22	88
Divorced	3	12
Occupation of the participants		
House wife	15	60
Farmer	5	20
Merchant	3	12
Daily worker	2	8
Education level of the participants		
No formal education	6	24
Elementary school(1–6)	11	44
Junior school (7–8)	5	20
High school(9–12)	3	12
Religion of the participants		
Protestants	22	88
Orthodox	2	8
Muslim	1	4
Number of children you have		
1	1	4
2–4	23	92
≥5	1	4

Three themes, 10 subthemes, and more than hundreds of codes were extracted after the interviews data were analyzed (Table 2). The main themes of this study were precipitators of early marriage, consequences of early marriage, and attitudes toward early marriage.

**Table 2:** Main themes and subthemes related to the effects of early marriage.

S. No	Main themes	Subthemes	Codes
1.	Precipitators of early marriage	1.1 Marriage by abduction 1.2 Arranged marriage	Abduction, girl’s parents, house band’s parent, house bands friends, neighbor’s women in relationship, relatives
2.	Consequences of early marriage.	2.1 Emotional damage 2.2 Moral damage 2.3 Early marriage is a risk factor for maternal and child health 2.4 Early marriage is a major cause for girls to drop out of school. 2.5 Poverty 2.6 Gender-based violence and divorce 2.7 Lack of essential life skills	Hopelessness, feeling of sadness, feeling of deep sorrow, helplessness, cried, suicidal ideation and attempt, went to grave, loneliness, suspicion, self-hate, feeling of remorse, humiliation, self-loathing, worthlessness, low self-esteem, guilt feeling poor pregnancy outcome(abortion, miscarriage), prolonged labor, cesarean delivery, lower abdomen pain, unplanned pregnancy, child death, frequent child illness, fail to resume education, fail to achieve objective, left bind class mates, unemployment, fail to meet basic needs, economic dependency, low social status, no communication with friends, not visits her parents, not participate in decision making, insult due to not give birth, strict control, physical abuse, dissolution of marriage, frequent conflict with husband, in ability to care baby, lack of skill to feed baby, in ability to manage marriage life
3.	Attitude toward early marriage	3.1 Early marriage is a harmful cultural practice; girls should be protected	individual women, society and country affect, it is like irreversible bone fracture, exposed to multiple problems, it brought unbearable failure, must be stopped, legal coverage to punish perpetrators

### Precipitators of early marriage

The participants in this study believed that the type of marriage practiced in the community and the people involved in early marriage aggravated the situation. This theme comprises 2 subthemes.

#### Marriage by abduction

Many participants mentioned that abduction was the major cause of early marriage.

*“My house band and his friend took me by force to his relative. I had no information about that… later they sent elders and reconciled with my parents” (a 25-year-old woman who was married at the age of 15).*

*“It was market day. While I went to the market, a group of people carried me forcefully and brought me to my husband’s parents” (a 24-year-old woman who was married at the age of 16).*

*“My husband had told me that he wanted to marry me. However, I was not willing to marry him. Suddenly one day he took me by force” (a 26-year-old woman who was married at the age of 16).*

#### Arranged marriage

The participants of this study pointed out that many people take part in early marriage. These include, the parents of the girls, the relatives, the neighboring women, the husband’s parents, and his relatives.

*“My husband’s neighbors misled me by telling me about his property. If I lost his marriage, they told me that I played with my fate” (a 25-year-old woman who was married at the age of 16).*

*“My parents told me and convinced me initially. Later, I was determined to marry him” (A 25-year-old woman, married at the age of 17).*

“*Neighbor women who were already married pushed me to marry at a young age. They wrongly informed me of the advantages of marriage at a young age” (a 25-year-old woman who was married at the age of 17).*

### Consequences of early marriage

All participants suggested that early marriage results in multifaceted negative effects on women’s lives. This theme consists of 7 subthemes.

#### Emotional damage

Almost all the women who participated in the interviews reflected that they were exposed to psychological problems as a result of their early marriage. They mentioned feelings of remorse, sadness, feelings of deep sorrow and crying, helplessness, suspicion, and suicidal attempts during marriage.

*“It is truly challenging time in my life that I was overwhelmed by feeling fear and worry about living with new family. Sometimes I cried” (a 26-year-old woman who was married at the age of 16).*

*“I was very sad. I cried every day, confused about what happened, I tried to flee from him and return to my parents’ home. There was a day when I tried to hang myself” (a 25-year-old woman married at the age of 15).*

*“When I started the housewife role, ever things could be new for me. I am excessively frustrated. I had obsessed with thoughts and cried” (a 23-year-old woman married at the age of 15).*

*“While I entered into marriage, everything went the opposite way for me. The way of life turned left, to be afraid, unreasonable anger and lack of self-confidence happened to me” (a 23-year-old woman married at the age of 15).*

*“I am surrounded by fear and suspicion, I think as I am going to die started from the day I entered into the marriage” (a 22-year-old woman married at the age of 15).*

#### Moral damage

The participants in the interviews also stated that many of them had suffered morally and faced feelings of humiliation, self-loathing, worthlessness, low self-esteem, and a sense of remorse during their marriage.

One participant mentioned, *“I doubted my humanity. I think as a person given for death or I think as a person went to a foreign country” (a 26-year-old woman married at the age of 16).*

Another interview participant said,*” The marriage life was new to me; I could not fulfill my responsibilities. I also felt regret and a lack of confidence” (an 18-year-old woman married at the age of 15)*.

#### Early marriage is a risk factor for maternal and child health

Most of the interview participants mentioned that early marriage is the main cause of maternal health problems, such as poor pregnancy outcomes, prolonged labor, operative delivery, chronic lower abdomen pain, difficulties during labor, serious illness during pregnancy, and chronic maternal health consequences following early marriage. Some of the participants responded as follows:

*“I had a hard time with my first delivery. I had encountered intense and longer labor pain. I gave my first birth by operation” (a 22-year-old woman married at the age of 15)*.

Another woman stated, “*After I suffered a long period of labor, I gave birth by cesarean section. I feel like the operative wound might be reopened. I do not move as I want due to fear of the operative site. I have encountered difficulty to providing care for my baby; especially the first birth because, I am inexperienced in looking after the baby and the operative wound, which is also another challenge*” *(A 26-year-old woman married at the age of 16)*.

Another woman also mentioned, *“The time of delivery was a very hard time for me. I gave my first and second births not in a natural way, but by cesarean section twice. Because of the surgery, I cannot move and do whatever I want. I think the knitted place may unravel” (a 22-year-old woman married at the age of 16)*.

“*I have challenged with health problems during pregnancy, and my first and second pregnancies were aborted two times*” *(a 24-year-old woman married at the age of 15)*.

*“My life is full of problems. My health is not in good condition. I have had four miscarriages. I always feel pain in my lower abdomen” (a 24-year-old woman married at the age of 15)*.

Another impact of early marriage is child health problems. The babies born to young adolescent mothers are frequently affected by illness and death. This was mentioned by a few participants as follows:

*“My children are not in good condition, their bodies are weak and they frequently have health problems and visit health facilities” (a 25-year-old woman married at the age of 17)*.

*“Early marriage is the sole reason for my current life. Health problems hurt my children frequently. I lost my first child due to illness” (a 24-year-old woman married at the age of 16).*

#### Early marriage is a major cause for girls to drop out of school

Early marriage occurs when girls are of high school age. For this reason, many girls who are married at the adolescence age are forced to drop out of school. After marriage, even if they are interested in continuing their studies, they are unable to resume education and fail to achieve their ambition. Many participants in the interviews affirmed that they dropped out of school because of early marriage. Some of the participants answered as follows:

“*When I entered into marriage, I was a grade five student. I cut my education due to marriage. My friends continued their education and reached a good level now. However, my life left behind” (a 25-year-old woman married at the age of 16).*

*“I was in grade eight when I entered into marriage due to the influence of my parents. I have not finished my studies and have not seen the fruits yet, always I worry about that” (a 26-year-old woman married at the age of 16).*

*“I was a high school grade nine student. I was forced into marriage by abduction. My plans to finish my studies and get where I wanted to go were not achieved” (a 25-year-old woman married at the age of 15).*

*“I was a grade ten student during my marriage. I am always bothered that I did not continue my education. I am very sad about the school dropout” (a 24-year-old woman married at the age of 16).*

#### Poverty

Early marriage occurs when couples have not obtained a permanent job, which means that they are economically dependent on their families. In this regard, most of the participants responded that poverty was a consequence of early marriage. The inability to meet their basic needs was mentioned by many participants. In addition, they reflect that the low social status and economic dependency that they are facing was the impact of their early marriage. Furthermore, they explained that they are not happy with their marriage life at all.

One participant stated, *“The standard of my life is too back and still; I had not come out of poverty. My children are in poverty, their bodies are weak and they frequently have health problems” (a 25-year-old woman married at the age of 17).*

Another participant said, *“I got married on my 16th birthday. Both of us were at a young age and did not have enough resources to sustain our lives. The reason for my poor current life is early marriage. I encountered health problems from time to time because of unfavorable conditions” (a 26-year-old woman married at the age of 16).*

Another woman also mentioned, *“Because the living conditions were not comfortable; health problems were hurting me and my children frequently. My living conditions are also backward. A shortage of money also affected my family” (an 18-year-old woman married at the age of 15).*

*“Being married at age 16 jeopardizes my life. It exposed me to poverty, daily labor, and a marriage breakdown” (a 24-year-old woman married at the age of 16).*

#### Gender-based violence and divorce

Participants in the interviews shared that early marriage is a cause of mistreatment, marriage insecurity, and the dissolution of marriage. Many participants answered that they were prevented from visiting their families, did not communicate with their friends, did not discuss their family’s issues, were insulted by their mother-in-law, and were physically abused by their husbands. Additionally, three women mentioned frequent conflicts and a lack of insecurity, and finally, they decided to divorce from their house bands. A few participants said the following:

*“I could not get pregnant and give birth as soon as I got married because I was too young. This made my husband and all his relatives hate and insult me. I was left alone. There was no one I could discuss with. When my husband beat me, there was no one to stop him” (an 18-year-old woman married at the age of 14).*

*“I felt lonely because I did not communicate with friends and I was under strict control. I have no role in deciding about my family. I was left doing only household chores. I am living in a stressful life” (a 24-year-old woman married at the age of 15).*

*“It was three years between marriage and my first pregnancy. This exposed me to insults and anger from his parents’ side. My husband said that he had a plan for another marriage if I did not give birth. I repeatedly thought about that. I was condemning myself” (a 23-year-old woman married at the age of 15).*

*“It rendered me to suffer. My marriage was broken up. We have four children. When the marriage was divorced, two of them were with me, and the remaining two remained with their father. I am currently raising two of my children and think of two children left with their father” (a 23-year-old woman married at the age of 15).*

*“It exposed me to great sorrow, and I still cry when I remember about it. It had an immense impact on me. During the marriage, both of us were mentally and physically immature enough. We were unable to handle conflict appropriately and my marriage was divorced” (a 25-year-old woman married at the age of 15).*

#### Lack of essential life skills

All the study participants stated that girls must have some essential life skills before entering marriage. Some participants in the interviews mentioned that they were too young during their marriage and were incapable of performing the duties and responsibilities of adult women. Few participants reflected on their experiences as follows:

*“I married at 16 years old. I have encountered difficulty in providing care for my baby, especially the first baby. Mother-in-law and Neighbor women discouragingly instruct me” (a 26-year-old woman married at the age of 16)*.

*“I got married on the 15th my birthday. When I started a housewife role, everything was new for me. I was frustrated; I had obsessed with thoughts and cried” (a 23-year-old woman married at the age of 15).*

*“Managing married life was difficult for me because of my inexperience. I could not fulfill my responsibilities and was vulnerable to negative criticism” (an 18-year-old woman married at the age of 15).*

### Attitudes toward early marriage

All the interview participants had negative attitudes toward early marriage. They strongly suggested that it should be avoided. This theme consists of 1 subtheme.

#### Early marriage is a harmful cultural practice; girls should be protected

All participants in this study affirmed that early marriage is a harmful cultural practice. They demonstrated the impact of early marriage which exposes young girls to serious problems that they cannot overcome. They said that girls should learn from their experiences and that they are willing to teach about the impact of early marriage on the future lives of girls. A few participants explained as follows:

*“I think early marriage is like an irreversible bone fracture. Its impact was serious. My life has not yet recovered” (a 26-year-old woman married at the age of 17).*

*“I think married early means surrendering yourself to suffering and playing on your future life. It has no advantage but is harmful. My whole life is empty because I have not reached my dreams” (a 25-year-old woman married at the age of 16).*

*“Early marriage is harmful. It seriously affected my life. I did not get a job and I was left behind in terms of education. It should receive legal coverage to be avoided, and all concerned bodies should act against it. Participants should be punished and girls should learn from us” (a 20-year-old woman married at the age of 16).*

## Discussion

The aim of this research is to examine the effects of early marriage on Guji women living in southern Ethiopia. The results of this study showed the factors that lead to early marriage and the various problems that resulted from it, putting women’s health and well-being in danger. One of the main themes of this study was the precipitators that led to early marriage. Early marriages were not solely driven by the partners’ desires. The current study indicated that marital culture, including abduction and arranged marriage, is precipitator of early marriage. Numerous participants repeatedly mentioned abduction and the involvement of both relatives and nonrelatives in their early marriage. This is due to the widespread belief that, despite legal prohibitions against it, abduction is a socially acceptable act and that getting married early is a great chance. These results are consistent with earlier research (Kok et al., 2023) showing early marriage to be an acceptable practice. These results, however, run counter to former study (Psaki et al., 2021) that identified the main causes of early marriage as poverty, a lack of opportunities, and pregnancy or the fear of becoming pregnant. This study revealed that adolescent girls are encouraged to pursue marriage by their neighboring women who are already married. This means that while creating intervention plans, planners of health programs and legislators should take into account many factors that contribute to early marriage.

Emotional and moral damage is another finding of this study. Following marriage, young women experience moral and psychological issues. This is due to a lack of mental maturity, inadequate stress management to handle marital difficulties, and taking an adult role. Likewise, prior studies have demonstrated that early marriage is linked to depression, stress, emotional pain, feelings of inferiority, low self-confidence, and regret (Yoosefi Lebni et al., 2023; Mrayan and Obeisat, 2021; Burgess et al., 2022; John et al., 2019). The results showed how important it is for women who were married before turning 18 to receive moral support and mental health interventions.

The interviewees reported that a large number of women had challenging problems in their marriages. Several individuals mentioned the following adverse consequences for maternal and child health: poor pregnancy outcome (abortion, miscarriage), unexpected pregnancy, extended labor, cesarean delivery, persistent lower abdominal pain, frequent child illness, and death. The pelvic bones of adolescent girls are immature enough to result in high obstetric risks, poor pregnancy outcomes, and complications during childbirth (Moerman, 1982). In a similar vein, prior research revealed that difficulties pertaining to pregnancy and labor, morbidity, and child mortality have been identified (Yoosefi Lebni et al., 2023; Mrayan and Obeisat, 2021; Baysak et al., 2021; Yaya et al., 2019; Adedokun et al., 2016; Dadras et al., 2023). This indicates that preventing early marriage is important for lowering the death and morbidity rates of mothers and children and that all relevant bodies should take this into consideration.

Early marriage interrupts a girl’s education. The study’s findings indicate that many participants drop out of school as a result of marrying. The reason for this discontinuation of education is that adolescent girls become pregnant, care for their child, and taking housewife responsibilities. Previous studies have shown that early marriage causes school dropout, negatively impacts girls’ academic prospects, and delays girls’ marriage are significantly associated with continuing their education (Mughal and Awan, 2020; Bengesai et al., 2021; Delprato et al., 2015). On the other hand, the present study revealed that despite their interest in continuing education after marriage, none of them returned to school. This suggests that for early married women to pursue their goals and further their education after marriage, they need all-encompassing support from their husbands, families, communities, and governmental and nongovernmental organizations.

One devastating effect of early marriage is poverty. This study indicated that several study subjects encountered difficulties in meeting basic needs, became economically dependent, and had low social status. The reason behind this is that girls marry early before obtaining permanent jobs. This could result in an economic dependency on their families and a shortage of income to sustain their lives. Previous research has demonstrated a significant association between poverty and early marriage (Dahl, 2010; Adekoya and Sokunbi, 2021). Delaying the marriage of girls can significantly aid in the achievement of sustainable development goals, especially in developing countries such as Ethiopia.

Another detrimental consequence of early marriage examined in this study is gender-based violence. Some participants stated that they were prevented from communicating with friends, did not visit their parents, did not participate in decision-making about their families, and were insulted due to not giving birth, being strictly controlled, and being physically abused. The reason for the strict control and physical mistreatment by husbands emanated from a wrong belief about married women the in study area in general, which was a strictly controlled woman during marriage became good housewife. Prior studies have shown that gender-based violence among women who marry early (Baysak et al., 2021; Yoosefi Lebni et al., 2023). This shows that the prevention of early marriage has a pivotal contribution to ensuring gender equality and avoiding mistreatment. Divorce has severe social repercussions, disrupts families, and endangers children (Damota, 2019). The results of this research demonstrate a link between early marriage and the dissolution of marriage. Likewise, prior studies have shown an association between early marriage and divorce (Tilson and Larsen, 2000; Alemu, 2008; Hunersen et al., 2021).

Essential life skills are a requirement for girls before marriage. Numerous research participants acknowledged in this study that they struggled to care for and feed their infants as well as to manage marriage life in general since they did not acquire basic life skills before becoming married. A study carried out in Jordan revealed that young married women have ambiguity regarding their roles as mothers (Al-Kloub et al., 2019). Another study conducted in Iran showed that early married women were not ready to accept marital and housewife roles (Kohan et al., 2021). Early married women should hence receive support from their families, neighbors, and community to help them acquire the necessary skills. Health care professionals such as health extension workers should also pay attention to detecting and educating young women about health issues.

All study participants had a negative attitude about young marriages. They thought it was a detrimental cultural practice that had an impact on the nation, society, and individual women. They view early marriage as a surrendering to suffering and liken its effects to an irrevocable bone fracture that is incapable of healing. Prior research conducted in Turkey and Jordan revealed that women who married at young age had unfavorable experiences in their marriage and negative perception on being married young (Baysak et al., 2021; Al-Kloub et al., 2019).

The study’s participants stated that they were prepared to take part in any initiative aimed at preventing early marriage. They intended to use their personal experiences to counsel young girls. This is a chance for all concerned bodies who want to participate in early marriage prevention programs.

### Strengths and limitations of the study

This study explored the effects of early marriage, which has received little attention globally, very few investigated in Ethiopia, and the findings could provide baseline information for the study area. In-depth face-to-face interviews were held in a separate room for the study to maintain participant privacy. This allowed the participants to openly share their experiences and permitted the collection of thorough data. On the other hand, this study has some limitations. First, women aged between 14 and 17 years were included in the study. It can be difficult to generalize the results to women who married under the age of 14. The study involved women who were married under the age of 18. As a result, information regarding the impact of early marriage on boys who marry before turning 18 cannot be provided.

## Conclusion

The present study’s findings illustrated the multidimensional effect of early marriage, necessitating the involvement of numerous sectors and stakeholders. The community, religious leaders, the health, education, and legal sectors, as well as the parents of young girls, should all take an active role in delaying early marriage. This indicates the development of policies that permit cooperation amongst all relevant parties.

The primary causes of early marriage in the research area were abductions and pressure from married neighboring women. Therefore, to develop culturally sensitive policies that involve early-married women in intervention plans for the sake of sharing their experiences with young girls; health program planners, policymakers, and all stakeholders should consider the factors that contribute to early marriage in the study area. The first steps should be taken to stop early marriage before it occurs. It is important to provide community-based education to increase community awareness of the negative effects of early marriage. Civic associations, educational institutions, and religious institutions should work against early marriage. To empower women, girls’ education must be promoted. Once early marriage occurs, risk mitigation should be the focus. To lessen the consequences, health professionals, particularly health extension workers, should focus on early-married women and offer health education. Women who marry at a young age lack fundamental life skills. Husbands, local ladies, and communities should therefore offer support. It is recommended that researchers use a quantitative longitudinal study design to ascertain the extent and trends of early marriage in the study area.

## Data Availability

The raw data supporting the conclusions of this article will be made available by the authors, without undue reservation.
